# Methamidophos poisoning: A paediatric case report

**DOI:** 10.1016/j.toxrep.2023.12.001

**Published:** 2023-12-07

**Authors:** Pierre de Villiers, Noor Parker, Roland van Rensburg, Pierre Goussard, Carine Marks, Veshni Pillay-Fuentes Lorente

**Affiliations:** a Department of Paediatrics, Faculty of Medicine and Health Sciences, Stellenbosch University, Cape Town, South Africa; b Division of Clinical Pharmacology, Department of Medicine, Faculty of Medicine and Health Sciences, Stellenbosch University, Cape Town, South Africa

**Keywords:** Organophosphate, Methamidophos, Paediatrics, Dermal absorption, Prolonged cholinergic syndrome

## Abstract

Methamidophos is a highly hazardous organophosphate and is known to cause an acute cholinergic toxidrome. Methamidophos use is not allowed in South Africa and therefore local data pertaining to methamidophos poisoning is very limited, with no paediatric clinical cases described. Methamidophos is an active metabolite of acephate, a commonly used organophosphate, registered for agricultural use in South Africa. We present a paediatric case of methamidophos poisoning with prolonged clinical effects. The patient experienced a prolonged cholinergic toxidrome lasting 10 days, with a period of near-full recovery during this time. We discuss the biological plausibility of the detected methamidophos being a byproduct of acephate. In addition, we highlight the importance of closer monitoring of patients with organophosphate poisoning in areas where acephate is commonly used.

## Highlights

• Organophosphate.
• Methamidophos.
• Paediatrics.
• Dermal absorption.
• Prolonged cholinergic syndrome.

## Graphical Abstract

**Figure:**
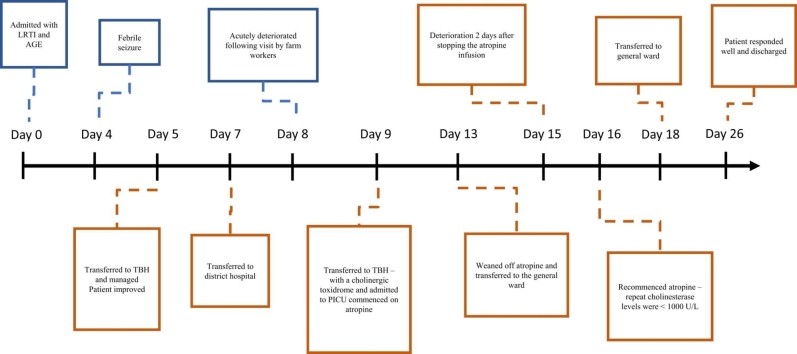

## 1 Introduction

Organophosphate poisoning remains a major public health issue in developing countries, including South Africa [1]. The majority of poisoning cases are due to household pesticides as opposed to agricultural pesticides [2], [3]. Most organophosphate poisoning cases are intentional and suicide related, as opposed to accidental poisoning [2]. Annually, an estimated 250 000–350 000 deaths are reported globally due to organophosphate poisoning, but the exact incidence is not known [2]. Likewise, the incidence of organophosphate poisoning among children is unknown. A retrospective review of the telephonic Tygerberg Poison Information Centre (PIC) consultations over a one-year period was conducted to determine the incidence of human poisoning in SA. Forty-four percent of cases included children under 13 years old. The survey found that pesticides were the most commonly reported non-drug chemical poisoning (34.8% of cases) [4]. A post mortem study conducted in the west metropole of Cape Town, South Africa, found that the most common organophosphates implicated in paediatric related deaths, include terbufos, methamidophos and diazinon [5]. The median age of decedents was 8.3 years [5]. We present a case of paediatric methamidophos poisoning with an unusually prolonged clinical syndrome following dermal exposure. To our knowledge this is the first clinical case description of methamidophos poisoning in South Africa.

## 2 Case

**Fig. 1:**
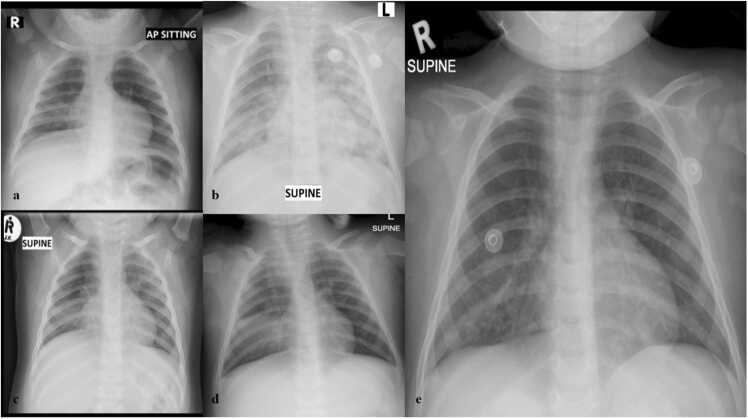
Chest X-ray findings: a) Chest x-ray at presentation: Right middle lobe changes present. b) Develops bilateral infiltrates in all the zones. c) X-rays showed improvement with the right middle lobe involved. d) Chest x-ray at PICU admission, shows progression of infiltrates again and consolidation in right middle lobe progressing. e) Chest x-ray at discharge from PICU shows significant clearing with mild right middle lobe changes.

A 15-month-old girl presented to a primary hospital within the Cape Town Metropole with features suggestive of a bronchopneumonia and acute gastroenteritis. Following confirmation of her diagnosis through radiological (see Fig. 1) and laboratory investigations she was commenced on ceftriaxone, nasal prong oxygen and intravenous fluids.

**Fig. 2:**
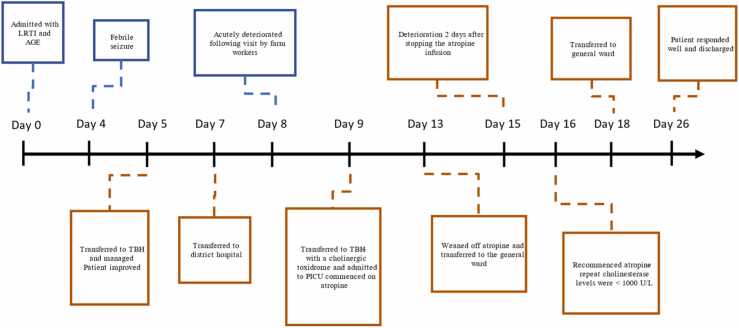
Timeline describing hospital admission. The blue blocks highlights that the events occurred in the primary hospital and orange blocks highlights the events occurred at the tertiary hospital.

However, on day 4 of admission she developed a febrile seizure and was referred to the tertiary hospital for further management. Further laboratory investigations were unremarkable and the patient improved on ceftriaxone. She was later transferred back to the primary hospital. At the primary hospital she deteriorated acutely with worsening respiratory distress, marked bronchial secretions and hypersalivation with an altered level of consciousness. A hospital-acquired infection was suspected and she was commenced on meropenem (antibiotic) and continuous positive airway pressure (CPAP) was applied. She was transferred back to the tertiary hospital. Fig. 2 depicts the patient’s clinical course.

On arrival at the tertiary hospital she was assessed as presenting with features suggestive of a cholinergic toxidrome. Her vital signs showed a respiratory rate (RR) 30 breaths per minute, pulse rate (PR) 150 beats per minute, blood pressure (BP) 128/63 mmHg, oxygen saturation (SATS) 96% (on CPAP with inspiratory concentration of oxygen of 40%) and temperature (T) 36.5 °C. Her Glasgow coma scale (GCS) was 8/15, pinpoint pupils and oral secretions noted with bilateral crepitations on chest auscultation. Following a detailed history, the mother reported that she and her work colleagues visited the patient at the primary hospital on their way home from work. They all worked with agricultural pesticides and had significant contact with the patient during the afternoon’s visiting hours. Four hours after visiting hours, the patient deteriorated. The patient was admitted to Paediatric Intensive Care Unit (PICU) with suspected organophosphate toxicity. Cholinesterase concentrations were < 1000 units per litre (U/L), confirming the likelihood of organophosphate toxicity. An atropine infusion was started at 0.05 mg/kg/hr with no initial bolus given. Blood culture during this admission was negative, and C-reactive protein (CRP) remained low. She was weaned off atropine by day 4 of PICU admission, based on resolution of secretions and bronchorrhea. She was transferred from PICU to the general ward the next day.

Repeat cholinesterase concentrations were 1140 U/L, five days after the initial cholinesterase concentrations and a day after discharge from PICU. Two days after stopping the atropine infusion, she had another acute deterioration suggestive of acute organophosphate toxicity. Her vital signs showed a RR: 48 bpm, PR: 178 beats per minute, BP: 146/95 mmHg, T: 39.0 °C and SATS: 96% on nasal prong oxygen (NPO2). Copious bronchial secretions and hypersalivation were noted with marked transmitted sounds and crepitations. The patient was lethargic but arousable, and pupils were constricted. Intravenous access was difficult and initially not obtained. Whilst awaiting PICU, the patient received three doses of intra-muscular atropine 0.2 mg, 15 min apart. She responded well with an improved level of consciousness, reduction in secretions and crepitations, but had a clinical seizure and required one dose of intramuscular lorazepam.

She was re-admitted to PICU and an atropine infusion was started via a right tibial intra-osseous line. Repeat cholinesterase concentrations were < 1000 U/L. Atropine was re-started at 0.05 mg/kg/hr, a blood and urine sample were taken for a toxicology screen and sent to the Division of Clinical Pharmacology at the University of Cape Town for analysis. The Sciex X500R was used and methamidophos was detected in both blood and urine samples at a peak intensity of 1.5e5 (blood) and 2.0e5 (urine), which is considered significant. Atropine was weaned as secretions and bronchorrhea allowed and stopped on day two of PICU re-admission. She made an uneventful recovery and was transferred to the general paediatric ward on day three of PICU re-admission. During her routine follow-up three weeks later at her local hospital, she was clinically well, and her repeat cholinesterase concentration was 5540 U/L.

## 3 Discussion

Organophosphates’ toxic effect in humans is primarily caused by blocking the function of acetylcholinesterase enzymes (AChE) [6], [7]. AChE’s function in the body is to break down acetylcholine (ACh) to choline and acetic acid. Organophosphate poisoning therefore leads to excessive amounts of ACh that bind to nicotinic and muscarinic receptors causing overstimulation of the different effector organs [6], [7]. Muscarinic overstimulation causes salivation, lacrimation, urination, defaecation, gastric emesis, bronchorrhea, bronchospasm and bradycardia. Nicotinic overstimulation causes fasciculations, muscle weakness and paralysis, lethargy, seizures and respiratory depression [7]. Organophosphates are readily absorbed through various routes, and exposures can occur through the oral, dermal or inhalation route. Atropine, a competitive, reversible, muscarinic receptor antagonist, alleviates muscarinic overstimulation and is the preferred treatment of organophosphate toxicity.

Organophosphate poisoning is a notifiable condition and in 2022, the National Institute for Communicable Diseases received 64 reported cases in the Western Cape province (estimated population size: 6.5 million). This equates to 0.98 cases per 100 000 population. Of the 64 reported cases, 33 were children 19 years and younger (51.5%). In our case we describe one of two possibilities: either the patient had exposure to acephate, a commonly used product in South Africa whose byproduct is methamidophos, or that the patient had direct exposure to methamidophos. According to the World Health Organization (WHO) classification of pesticides, methamidophos is regarded as a highly hazardous (class Ib) organophosphate[8]. Methamidophos poisoning is a rare occurrence and the true incidence in South Africa is not known. Of 54 pesticide-related deaths over a ten year period in Cape Town’s west-metropole, only two cases were attributed to methamidophos poisoning, which only account for 0.0004% of unnatural deaths during this period [5]. Low-dose exposure of methamidophos in agriculture is a well described phenomena with various long-term sequela, and as such has been banned as a commercial agricultural pesticide in various countries, including South Africa hence making the second possibility unlikely [9], [10]. Acute methamidophos poisoning is rare in our setting with very few described cases.

Acephate is an organophosphate compound that is registered for agricultural use in South Africa and is readily used on vineyards. [11]. It is classified as a moderately hazardous compound according to the WHO [8]. However, acephate itself is a weak acetylcholinesterase inhibitor (human toxicity threshold of 1030 mg/kg), but the active metabolite, methamidophos, is a potent acetylcholinesterase inhibitor [12] with a human toxicity threshold of 13 mg/kg [12], [13]. As acephate is a registered pesticide in South Africa, and as the farmworkers worked on a commercial farm there is a high probability that acephate and not the illegal methamidophos was used. Methamidophos was detected in the plasma of the patient whereas acephate was not. Therefore, while acephate was the likely primary exposure, the severity, protractedness, and biochemical confirmation make methamidophos the likely culprit as a by-product of acephate.

Our patient’s exposure to organophosphates likely occurred while being in the primary hospital. It is reasonable to consider that the exposure happened when the mother and her co-workers visited the patient during afternoon visiting hours. They work with pesticides in vineyards and were returning home after work. With no oral exposure being noted, the most likely route of exposure was dermal. The source is likely to be the workers’ clothes. Acephate in itself can lead to mild to moderate cholinergic symptoms, but as it is metabolized to methamidophos, the cholinergic syndrome will continue and even worsen as methamidophos is a more potent acetylcholinesterase inhibitor [12], [13]. Low dose dermal exposure in humans lead to effects within 30 min to 8 h post exposure [14], whereas high dose exposure in rats, have a peak blood concentration after 1–3 h post exposure [12]. The patient developed symptoms 4 h after visiting hours, which corresponds with dermal exposure’s timeline of exposure to effect. Natural degradation of acephate occurs over a couple of days, therefore the acephate on the workers’ clothes during visiting ours would still be toxic [10], [15]. After in depth discussions with the family it was clear that no organophosphate-like substance was given to the patient at home or in hospital.

Skin ageing affects absorption of compounds, especially hydrophilic agents, such as acephate. As skin ages the surface lipid content diminishes and skin hydration is reduced, leading to lower percutaneous absorption of hydrophilic agents [16]. Prolonged sun exposure, as is often seen in farm workers, leads to acceleration of skin ageing [17]. A 15-month-old child, on average, has a skin surface area two times greater, by volume, when compared to an adult. The amount of absorption, by body volume, is far greater than in an adult [18]. Chronic exposure to low doses of organophosphorus insecticides, leads to down regulation of the muscarinic cholinergic receptors, inducing the development of tolerance to the toxic effects of organophosphorus insecticides [19]. All of the above could explain why the mother and her co-workers were asymptomatic, but our patient developed severe symptoms.

Animal studies have showed that acephate has a multiphasic elimination from plasma, with rapid elimination during the first two hours with a half-life of 1.4 h, but with terminal elimination starting at 24 h with a half-life of 50 h [20]. Acephate is widely distributed in plasma with the highest distribution noted at 0.5 – 1 h after dosing, especially in well perfused organs such as the heart, brain and liver[20]. Elimination of acephate is largely through excretion in urine (83–89% of administered dose) [20]. Methamidophos, a breakdown product of acephate, accounted for 5% of urine collection following acephate exposure [20].

Observations in humans, when using acephate at a dose up to a hundred fold smaller than that used in animals, showed similar pharmacokinetics [20]. Time to peak tissue concentration (Tmax) was 1–4 h after administration and plasma concentration measurements at 24 h revealed concentrations of < 6% of maximum serum concentration (Cmax) and undetectable at 48 h [20]. A sex-difference of acephate disposition was demonstrated with 48 h urine collections recovering 26–62% of the administered dose in males and 12–53% in females [20]. Methamidophos accounted for 1.3% of the amount recovered in the urine [20]. Most of the recovered acephate and methamidophos were found during the first 12 h after administration [20].

When small doses of acephate are ingested, used in toxicity studies in humans, there are no evidence of any persistent accumulation in tissues [20]. When larger doses are ingested, used in toxicity studies in animals, tissue accumulation occurs and is proportional to the dose received [20]. Our patient likely had high dose exposure to acephate leading to acute symptoms. As the acephate accumulated in tissues and underwent further metabolism, the active metabolite methamidophos was produced, leading to ongoing symptoms.

A differential diagnosis in our case could be the intermediate syndrome. Intermediate syndrome presents with distinct neurological symptoms appearing 24–96 h after exposure and resolution of cholinergic symptoms [21], [22], [23]. The major symptoms include neck flexion weakness, proximal muscle weakness, respiratory insufficiency, decreased deep tendon reflexes and cranial nerve fall outs [21], [23]. Literature is unclear whether fat solubility plays a role in the incidence of intermediate syndrome, but some authors have suggested that lipophilic organophosphates lead to an increase incidence of intermediate syndrome [21], [24]. Prolonged cholinergic syndromes are well described in the literature, albeit rare, and are usually caused by lipophilic agents, such as malathion, but methamidophos is hydrophilic [15], [25]. In our case intermediate syndrome was not considered as the patient did not present with typical clinical features.

Importantly, organophosphate agents lead to diverse symptomology and respond differently to oximes and anti-cholinergic antidotes, suggesting that each organophosphate substance should be treated and reviewed independently [25].

## 4 Conclusion

To our knowledge this is the first case to highlight the prolonged cholinergic toxidrome of methamidophos poisoning in a paediatric patient. Although a prolonged toxidrome is well described in the literature, paediatric cases are rarely encountered in practice. This case recreates awareness for clinicians to closely monitor children following organophosphate poisoning especially in areas with acephate use. In addition, community education is needed to limit the spread of organophosphate exposures through dermal contact by workers, particularly to children, in communities.

## 5 Learning points

• Even though it is rare, a prolonged cholinergic toxidrome does occur with organophosphates, calling for meticulous monitoring once acute symptoms have improved or resolved.
• Ongoing public education and awareness are required regarding the dangers of organophosphates and possibility of toxicity secondary to dermal exposure, especially in children.

## Funding

This research did not receive any specific grant from funding agencies in the public, commercial, or not-for-profit sectors.

## CRediT authorship contribution statement

**Parker Noor:** Supervision, Writing – review & editing. **de Villiers Pierre:** Writing – original draft, Writing – review & editing, Conceptualization, Data curation, Formal analysis, Funding acquisition, Investigation, Methodology, Project administration, Resources, Software, Validation, Visualization. **Pillay-Fuentes Lorente Veshni:** Supervision, Writing – original draft, Writing – review & editing. **Marks Carine:** Supervision, Writing – review & editing. **Goussard Piere:** Supervision, Writing – review & editing. **Van Rensburg Roland:** Supervision, Writing – review & editing.

## Declaration of Competing Interest

The authors declare that they have no known competing financial interests or personal relationships that could have appeared to influence the work reported in this paper.

## Data Availability

Data will be made available on request.
